# Investigating the role of UBASH3B in cancer: structural relevance, physiological functions, and therapeutic possibilities

**DOI:** 10.1186/s13046-025-03511-7

**Published:** 2025-08-30

**Authors:** Sophie Werner, Cameron Westlake, Madeleine Ndahayo, Ishita Gupta, Daria A. Gaykalova

**Affiliations:** 1 Institute for Genome Sciences, University of Maryland School of Medicine, 670 West Baltimore Street, Baltimore, MD 21201 USA; 2 Department of Otorhinolaryngology-Head and Neck Surgery, Marlene & Stewart Greenebaum Comprehensive Cancer Center, University of Maryland Medical Center, MD 21201 Baltimore, USA; 3 Department of Oncology, Sidney Kimmel Comprehensive Cancer Center, Johns Hopkins University, MD 21231 Baltimore, USA

**Keywords:** HNSCC, UBASH3B, EGFR, TULA2, STS-1, Oncogene, Therapeutic targets

## Abstract

Head and neck squamous cell carcinoma (HNSCC) is the seventh most common cancer globally and presents a persistent clinical challenge due to the limited availability of effective targeted therapeutics. Recent studies have identified the ubiquitin-associated and SH3 domain-containing B (UBASH3B), a tyrosine phosphatase, as a key oncogenic player in HNSCC pathogenesis. Elevated UBASH3B expression correlates with poor clinical outcomes in HNSCC patients. Mechanistically, UBASH3B promotes tumor progression by stabilizing the epidermal growth factor receptor (EGFR) levels, thereby enhancing downstream signaling pathways that promote cancer cell proliferation, survival, and therapeutic resistance. In this review, we provide a comprehensive overview of the structural features and physiological functions of UBASH3B, along with a focused discussion on its emerging role in HNSCC tumorigenesis. We further explore the potential of targeting UBASH3B as a novel therapeutic target, underscoring its promise in reshaping treatment paradigms. Elucidating the molecular functions of UBASH3B in HNSCC may uncover new vulnerabilities and pave the way for the development of novel therapeutic strategies that target its activity.

## Introduction

Head and neck squamous cell carcinoma (HNSCC) encompasses cancers of the epithelial lining of the oral cavity, pharynx, or larynx [1]. As of 2020, it is the seventh most common cancer worldwide, with about 890,000 new cases and 450,000 deaths yearly [2]. HNSCC remains a significant health concern, with approximately 54,000 new cases diagnosed and 11,230 deaths reported in the United States in 2022 [3]. HNSCC represents about 3% of all cancers and 2% of all cancer-related deaths. HNSCC predominantly affects men, with an incidence of 17.2 per 100,000, particularly among non-Hispanic White and American Indian/Alaska Native populations. In women, the rate is nearly three times lower [4]. Statistics indicate that only 27% of cases are detected at a localized stage, while 51% are locally advanced and 15% are metastatic at diagnosis [4]. The 5-year survival rate is 68.5%, with outcomes heavily dependent on disease stage—86.6% for localized, 69.1% for locally advanced, and 39.3% for metastatic disease [4]. Since 1975, mortality has decreased by 44%, with survival improving from 54.6 to 68% by 2018, driven by advances in screening, surgery, radiation, and immunotherapy [4]. Additionally, this improvement is predominantly attributed to HPV-positive (HPV+) cases, which now make up approximately 25% of HNSCCs, particularly oropharyngeal squamous cell carcinoma (OPSCC) [5]. HPV + tumors are biologically distinct from HPV-negative (HPV-) HNSCC, differing in genetic, epigenetic, and clinical characteristics [6]. While treatment strategies remain similar, survival outcomes are starkly different; as the 5-year overall survival for HPV- HNSCC remains poor (25–40%), whereas HPV + patients demonstrate significantly better survival rates (80%), regardless of stage [6, 7]. Most HNSCC cases (75%) are associated with tobacco and alcohol use [8]. Other factors include environmental pollutants, poor oral hygiene, aging, and a diet lacking fruits and vegetables [8, 9]. Regional variations in risk factors exist, such as declining tobacco use in high-income countries, which has contributed to a shift in HNSCC etiology, with HPV-associated cases increasing, especially in the U.S. and Western Europe [10]. Despite medical advances, discrepancies in survival rates exist due to disparities in access to care that persist, particularly among racial minorities and underserved or socioeconomically disadvantaged communities, suffering a greater incidence and lower overall survival [11].

HNSCC of the oral cavity is typically managed based on the stage through surgical resection followed by adjuvant chemoradiation or radiation [12]. Chemoradiation is also the standard treatment for cancer originating in the pharynx or larynx [13]. While early-stage oral cavity cancers may be treated with surgical resection alone and laryngeal cancers with surgery or radiation, the majority of HNSCC cases demand a multimodal approach, necessitating multidisciplinary care [14]. Cetuximab, an epidermal growth factor receptor (EGFR) monoclonal antibody, is FDA-approved as a radiosensitizer for recurrent or metastatic disease, either alone or in combination with chemotherapy [15]. Additionally, immune checkpoint inhibitors nivolumab and pembrolizumab, both targeting PD-1, are FDA-approved for treating cisplatin-refractory recurrent or metastatic HNSCC, with pembrolizumab also approved as first-line therapy for unresectable or metastatic cases [16–18]. Molecular characterization and immune profiling of HNSCC suggest that integrating prognostic and predictive biomarkers into clinical management may enhance the effectiveness of targeted therapies and improve patient survival outcomes.

Genetic alterations (mutations, amplifications, and alterations in the expression of genes) play a crucial role in the development and progression of HNSCC, influencing tumor behavior, treatment response, and patient outcomes [19, 20]. High-throughput sequencing has illuminated the genetic landscape of HNSCC, revealing distinct mutation patterns in HPV + and HPV- tumors [21–24]. Although HPV + cases show fewer mutations, TCGA data indicate a narrower difference [21–24]. Notably, HPV- tumors with lymph node metastasis exhibit higher mutation burdens, linking mutational load with tumor aggressiveness and highlighting molecular differences influencing prognosis and progression [25]. The genetic mutations and epigenetic alterations underlying HNSCC differ based on their HPV status. HPV- tumors tend to exhibit a significantly higher rate of somatic mutations compared to their HPV + counterparts. This disparity is mainly attributable to the underlying carcinogenic mechanisms. In HPV- HNSCC, the elevated mutational burden is strongly associated with tobacco exposure, where carcinogens cause extensive DNA damage, leading to genomic instability and mutation-driven tumor development [26]. Conversely, HPV + HNSCC arises from viral oncogenesis, primarily through high-risk HPV types expressing E6 and E7 proteins, which inactivate the tumor suppressors p53 and pRB.

The mutational landscape of HNSCC highlights key differences between HPV + and HPV- disease. In HPV- HNSCC is driven by deletions in cell cycle regulatory genes (CDKN2A, CCBD1, TP53), mutations in WNT signaling genes (FAT1, AJUBA, NOTCH1), mutations in epigenetic regulation genes (KMT2D, NSD1), and amplification of cell growth genes (EGFR), many of which are also implicated in other malignancies linked to tobacco use [21–23]. These mutations primarily affect pathways governing cell cycle control, leading to unchecked cell proliferation and enhanced susceptibility to malignant transformation [21, 27]. Conversely, in HPV + HNSCC, overexpression of E6 and E7 oncoproteins inhibits apoptosis and promotes viral replication, respectively contributing to carcinogenesis [28]. Frequently mutated genes in HPV + HNSCC include PIK3CA, DDX3X, FGFR2, FGFR3, KRAS, MLL2/3, and NOTCH1 [21–23]. In HPV + tumors, PIK3CA frequently harbors hotspot mutations (E542K, E545K) from C > T transitions, promoting oncogenic signaling through pathways such as PI3K/Akt/mTOR [29]. On the other hand, HPV- tumors show more dispersed PIK3CA mutations [29]. Similarly, the tumor suppressor TP53 shows divergent mutation patterns. In HPV + HNSCC, TP53 is rarely mutated due to the functional inactivation of p53 by the viral E6 protein [21]. In contrast, TP53 mutations are highly prevalent in HPV tumors, with mutations occurring randomly throughout the gene, contributing significantly to impaired DNA repair, loss of cell cycle control, and increased genomic instability [21]. These differences extend into the mutational landscape of recurrence and metastasis. Recurrent HPV + tumors tend to accumulate new TP53 mutations, while PIK3CA mutations become less frequent compared to primary HPV + tumors, thus indicating a dynamic shift in genetic drivers as the disease evolves [30]. In contrast, HPV recurrent tumors often maintain their high mutational burden. Whole-exome sequencing studies of synchronous nodal metastases and metachronous recurrences revealed novel mutations in C17orf104 and ITPR3 in nodal metastases, while DDR2 mutations emerged in recurrent lesions [25], highlighting the ongoing genetic diversification associated with tumor progression. Notably, Fanconi anemia, a rare inherited genetic disorder caused by mutations in FANC genes, impairs DNA repair and significantly increases the risk of developing HNSCC [31]. Polymorphisms in genes involved in carcinogen metabolism and immunity, including CTLA4, IL10, CYP1A1, and GSTM1, are also associated with an increased risk of developing HNSCC [32–35].

Intriguingly, alterations in EGFR signaling are more frequent in HPV- HNSCC (15%) as compared to HPV + tumors (6%) [21]. EGFR, a prominent oncogene in HNSCC, is overexpressed in approximately 30% of HNSCC cases [36, 37] and is frequently linked with the upregulation of vascular endothelial growth factor (VEGF), particularly in HPV- cases [38]. Additionally, EGFR overexpression serves as a negative prognostic marker, correlating with reduced overall survival (OS), disease-free survival (DFS), and earlier relapse [39, 40]. EGFR functions as a transmembrane protein that heterodimerizes upon ligand binding, triggering a conformational change that activates its intrinsic kinase activity. This results in autophosphorylation and recruitment of downstream effectors, activating a number of signaling pathways including the Ras/MAPK pathway, PI3K/Akt pathway, and the Jak/STAT cascade, which play pivotal roles in cellular proliferation, survival, invasion, and metastasis, while simultaneously reducing apoptosis [41]. Regulation of EGFR is crucial for preventing pathological signaling and is primarily controlled through receptor endocytosis, a process mediated by the adaptor protein GRB2 and the ubiquitin ligase c-CBL, which facilitate EGFR internalization and subsequent degradation [42]. Emerging evidence suggests that ubiquitin-associated SH3 domain-containing B (UBASH3B) is a key modulator of EGFR signaling. UBASH3B dephosphorylates CBL, which leads to EGFR endocytosis and degradation [43]. Dysregulation of UBASH3B has been implicated in enhancing EGFR-mediated oncogenic signaling, suggesting a plausible role underlying the aggressive phenotype and poor clinical outcomes associated with HNSCC.

UBASH3B belongs to the UBASH3 family of protein tyrosine phosphatases, which consists of UBASH3A and UBASH3B [44]. Both proteins share a similar structure of organization, comprising a ubiquitin-associated (UBA) domain, an Src-homology 3 (SH3) domain, and a histidine phosphatase domain, although they differ slightly in sequence [44]. Members of this group play diverse roles in immunity, cancer, and development, earning them multiple aliases. UBASH3A is also known as suppressor of T cell signaling 2 (STS2), T cell ubiquitin ligand 1 (TULA1), and CBL-Interacting Protein 4 (CLIP4), while UBASH3B is alternatively known as suppressor of T cell signaling 1 (STS1), T cell ubiquitin ligand 2 (TULA2), and p70 [44]. UBASH3 proteins are also implicated in regulating inflammatory responses by modulating cytokine production and the activation state of immune cells, thereby shaping the inflammatory microenvironment [45]. Emerging evidence suggests that UBASH3 proteins may influence cell fate decisions or signaling events critical for embryonic development [46], although the underlying mechanisms remain nascent.

Recent studies have linked UBASH3B dysregulation to cancer progression and proliferation. Specifically, overexpression of UBASH3B has been shown to promote invasion and metastasis in triple-negative breast cancer and enhance proliferation in acute myeloid leukemia [47, 48]. These effects are mediated through the dephosphorylation of CBL by UBASH3B; dysregulated UBASH3B activity disrupts CBL’s function, although the downstream effects of CBL inactivation differ between each cancer type. Furthermore, the ability of UBASH3B to influence signaling pathways, such as those related to cell proliferation, angiogenesis, and immune regulation, underscores its critical role in oncogenesis.

In this review, we aim to provide a comprehensive analysis of the different roles of UBASH3B in cancer initiation and progression, as well as its potential role in HNSCC, along with a focus on therapeutic responses.

**Fig. 1:**
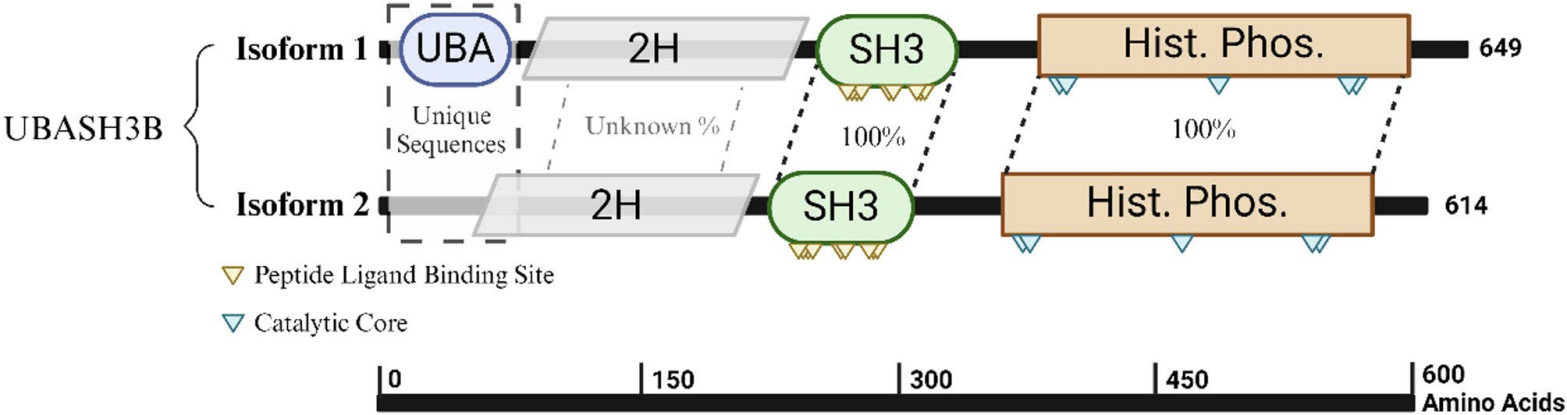
Schematic representation of UBASH3B protein structure. **UBASH3B** gene encodes two transcription isoforms with distinct domain compositions. Isoform 1 is the full-length protein, comprising 649 amino acids and 4 distinct domains: a N-terminus ubiquitin-associated (**UBA**) domain, a 2 H phosphodiesterase domain, a central Src homology 3 (**SH3**) domain, and a C-terminal histidine phosphatase domain, which contains the active site for **UBASH3B’s** tyrosine phosphatase activity. On the other hand, isoform 2 is shorter, with a length of 614 amino acids, and differs primarily near the N-terminus. The truncated isoform 2 entirely lacks the **UBA** domain, and it remains unclear where the 2 H phosphodiesterase domain is retained. Sequence alignment shows 100% identity between the two isoforms beginning from the 72nd amino acid of isoform 1 and the 37th amino acid of isoform 2, indicating a shared sequence downstream of the **UBA** region. Figure created with BioRender.com

The UBASH3B gene is located on chromosome 11 (11q24.1 from base pair 122,655,722 to 122,814,473), encompassing approximately 6,865 kb of DNA. UBASH3B’s RNA is composed of 14 exons. UBASH3B’s protein consists of 649 amino acids and has a molecular mass of 74,123 Da (Fig. 1) [49].

UBASH3B is a multifunctional protein with distinct domains that are essential for its regulatory roles in cellular signaling. UBASH3B comprises four primary domains, namely, an N-terminal ubiquitin-associated domain (UBA), a 2 H phosphodiesterase domain that is structurally fused to the central Src homology 3 (SH3) domain, plus the active site at the C-terminal histidine phosphatase domain (Fig. 1) [50]. The N-terminus SH3 and UBA domains are responsible for mediating protein-protein interactions with lengths of 61 amino acids and 37 amino acids, respectively (Fig. 1). The UBA domain serves as a ubiquitin receptor by binding to ubiquitinated proteins such as Aurora B and CBL, which are commonly overexpressed in HNSCC [51]. Through these interactions, the UBA domain plays a critical role in regulating the expression and activity of these proteins, thereby contributing to tumor suppression and the modulation of oncogenic pathways. Structurally interposed between the UBA and SH3 domains is the 2 H phosphodiesterase domain (Fig. 1), a conserved yet poorly characterized module. Though its precise enzymatic function remains fully elucidated, the domain is postulated to interact with the C-terminal histidine phosphatase domain to hydrolyze phosphoesters during dephosphorylation processes in various substrates [50, 52]. Its positioning suggests a scaffold-like role that may support conformational integration of the protein’s N-terminal interaction domains with its C-terminal catalytic machinery, thus enabling UBASH3B to function as a dynamic regulator of signaling cascades. Following the 2 H phosphodiesterase domain lies the SH3 domain, which recognizes and binds to proline-rich sequences in target proteins, such as the proline-rich region of CBL, facilitating the assembly of protein complexes essential for EGFR signaling and other transduction pathways, demonstrating a link between UBASH3B and EGFR signaling [53]. Together, the SH3 and UBA domains enable UBASH3B to orchestrate intricate protein interactions and regulatory mechanisms crucial for maintaining cellular homeostasis and controlling cancer development. The C-terminal histidine phosphatase, the largest domain at approximately 212 amino acids, plays a central role in UBASH3B’s high phosphatase activity. This domain contains the active site for UBASH3B’s tyrosine phosphatase activity, which is essential for its regulatory functions in various signaling pathways. The domain is made up of a central catalytic region with two histidine residues and two highly conserved arginine residues (Fig. 1). Similar to other PGM-family histidine phosphatases, the phosphatase activity of this domain in UBASH3B involves a phosphoryl transfer to one of the histidine phosphatases while the other residues are hydrogen bonded to the phosphate group throughout the transfer [54]. The phosphate transfer is initiated by a nucleophilic attack by the first conserved histidine residue, followed by hydrolysis of the histidine intermediate [54]. This domain is directly involved in the dephosphorylation of the EGFR at multiple tyrosine kinases, as well as the inhibition of T-cell receptor (TCR) signaling via ZAP-70 dephosphorylation, thereby regulating the signaling pathways involved in cell proliferation and immune responses [55, 56].

The spliced variant of UBASH3B, isoform 2, lacks the UBA domain entirely, and it remains unclear whether this short isoform retains the 2 H phosphodiesterase domain (Fig. 1) [57]. This structural variation could significantly alter the isoform’s protein-protein interaction capacity and phosphatase activity. While the functional consequences of this isoform have not been previously investigated, the absence of the UBA domain likely impairs critical functions of UBASH3B [57]. Insights into these potential functional losses could be gleaned from studies on UBASH3A, a homolog with similar domain architecture and function. Deletion of the UBA domain in UBASH3A completely inhibited its ability to bind to ubiquitinated proteins [53]. Given the shared functional properties of the UBA domains in UBASH3A and UBASH3B, it is plausible that UBASH3B isoform 2 would exhibit similar functional deficiencies, potentially compromising its role in modulating signaling pathways and ubiquitin-mediated regulation [53].

The following section will discuss the function of UBASH3B under normal conditions and during oncogenic transformation.

## Physiological functions of UBASH3B

### Normal cells

UBASH3B is a multifaceted phosphatase and acts primarily as a protein tyrosine phosphatase (PTP) while also exhibiting non-PTP functions that contribute to maintaining homeostasis, extending beyond its well-characterized functions in the immune system. UBASH3B. In the hematopoietic system, UBASH3B dephosphorylates key receptor tyrosine kinases, FLT3 and KIT, which are essential for the maintenance and proliferation of hematopoietic stem and progenitor cells [58]. Loss of UBASH3B function enhances the proliferation of these cells, suggesting its role as a negative regulator of hematopoietic homeostasis [58]. Similarly, in the skeletal system, UBASH3B-mediated dephosphorylation of spleen tyrosine kinase (Syk) is implicated in inhibiting osteoclast formation, thereby contributing to the regulation of bone density and health [59].

Within the immune system, UBASH3B serves as a critical regulator of both the innate and adaptive immune systems, modulating signaling pathways in T cells, B cells, mast cells, and platelets. In T cells, UBASH3B negatively regulates T cell receptor (TCR) signaling by dephosphorylating ZAP-70, a key kinase involved in early TCR activation, thereby preventing aberrant T cell activation and maintaining immune homeostasis [60]. In B cells and other lymphoid cells, UBASH3B interacts with Syk to suppress downstream signaling events, including those involved in the PI3K-mTOR signaling pathway, while concurrently enhancing JAK1-STAT1 signaling by repressing CBL. This dual regulatory mechanism is linked to increased autophagic activity, underlining the role of UBASH3B in balancing immune cell metabolism and function [61, 62]. In platelets, Zhou and colleagues [63] reported that UBASH3B enhances the negative regulation of FcγRIIA signaling by dephosphorylating Syk, ultimately reducing thrombosis. This process was regulated post-transcriptionally by miR-148a, which targets UBASH3B, and inhibition of miR-148a increases UBASH3B levels and promotes its antithrombotic effect [63].

UBASH3B also modulates effector functions in innate immune cells. In mast cells, UBASH3B regulates FcϵRI signaling by modulating Syk phosphorylation, thereby preventing degranulation and inflammation [64]. Likewise, in platelets, UBASH3B inhibits glycoprotein VI (GPVI)-mediated activation by dephosphorylating Syk, consequently reducing platelet activation and thrombus formation [65]. Consistent with this, mice deficient in UBASH3B exhibit shorter bleeding times, reflecting a hypercoagulable state due to increased clot formation [66].

Collectively, these findings underscore the broad biological relevance of UBASH3B in maintaining systemic homeostasis. Its ability to regulate tyrosine kinase activity in diverse signaling contexts highlights its potential as a key modulator in hematopoiesis, immunity, inflammation, and thrombosis. Given its widespread regulatory functions, research is exploring the role of UBASH3B in cancer cells, where it can influence oncogenic signaling pathways and tumor progression.

### Cancer cells

**Fig. 2:**
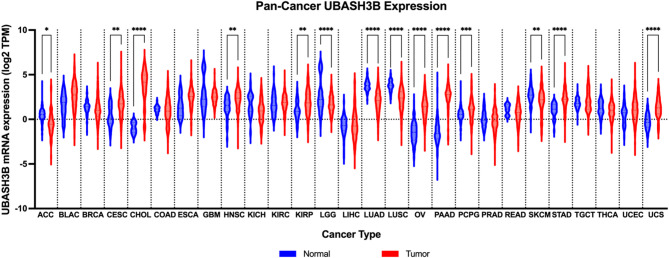
Transcriptional Expression Analysis of UBASH3B in Pan-cancer. The violin plot illustrates the transcriptional expression analysis of **UBASH3B**using data from the OncoDB database [67], in the 27 cancer types analyzed between the normal and tumor tissue samples. The x-axis denotes the tissue type, while the y-axis indicates the relative expression level of UBASH3B. Statistical significance is denoted by asterisUBASH3Bks: *p < 0.05, **p < 0.01, ***p < 0.001, ****p < 0.0001. N: Normal tissue; T: Tumor tissue. **ACC**: Adrenocortical carcinoma; **BLCA**: Bladder Urothelial Carcinoma; **BRCA**: Breast invasive carcinoma; **CESC**: Cervical squamous cell carcinoma and endocervical adenocarcinoma; **CHOL**: Cholangiocarcinoma; **COAD**: Colon adenocarcinoma; **ESCA**: Esophageal carcinoma; **GBM**: Glioblastoma multiforme; **HNSC**: Head and Neck squamous cell carcinoma; **KICH**: Kidney Chromophobe; **KIRC**: Kidney renal clear cell carcinoma; **KIRP**: Kidney renal papillary cell carcinoma; **LGG**: Brain Lower Grade Glioma; **LIHC**: Liver hepatocellular carcinoma; **LUAD**: Lung adenocarcinoma; **LUSC**: Lung squamous cell carcinoma; **OV**: Ovarian serous cystadenocarcinoma; **PAAD**: Pancreatic adenocarcinoma; **PCPG**: Pheochromocytoma and Paraganglioma; **PRAD**: Prostate adenocarcinoma; **READ**: Rectum adenocarcinoma; **SKCM**: Skin Cutaneous Melanoma; **STAD**: Stomach adenocarcinoma; **TGCT**: Testicular Germ Cell Tumors; **THCA**: Thyroid carcinoma; **UCEC**: Uterine Corpus Endometrial Carcinoma; **UCS**: Uterine Carcinosarcoma

Recent studies have linked UBASH3B to tumor progression, immune evasion, and therapeutic resistance in several cancer types. UBASH3B exhibits dual expression patterns across multiple cancer types in the Pan-cancer analysis, functioning either as a tumor suppressor or oncogene, depending on the tumor type (Fig. 2). The following sections will discuss the role of UBASH3B in contributing to tumor growth and metastasis.

**Fig. 3:**
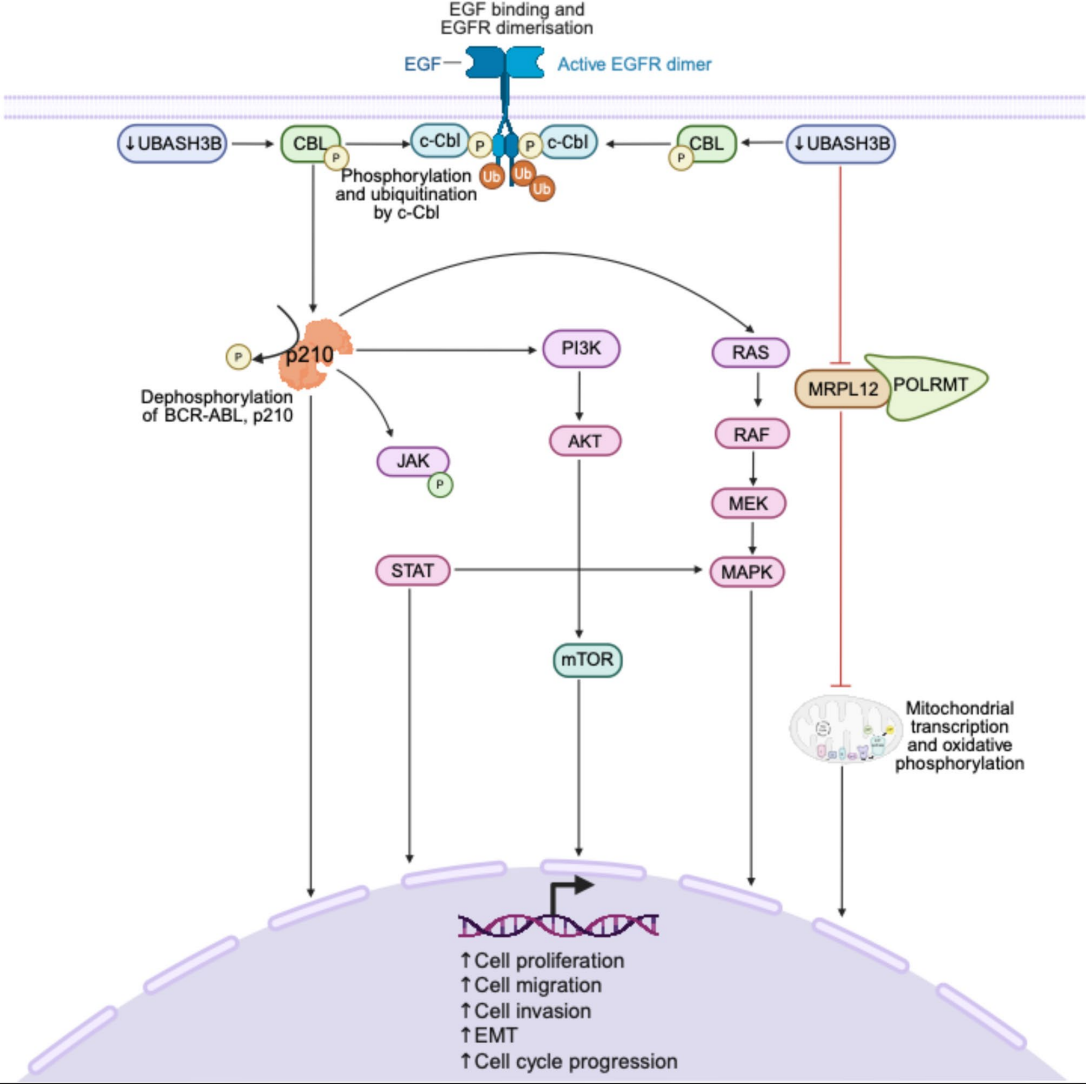
UBASH3B mediated tumor suppressive signaling. Loss of **UBASH3B** leads to increased phosphorylation of **CBL**, relieving its inhibition and promoting receptor tyrosine kinase (**RTK**) signaling. **UBASH3B** deficiency dephosphorylates the **BCR-ABL** fusion protein (**p210**), contributing to oncogenic signaling in leukemia. The **p210** fusion protein also triggers downstream signaling pathways, including **JAK/STAT**, **PI3K/Akt/mTOR**, and **Ras/Raf/MEK/ERK**. **UBASH3B** loss also disrupts its interaction with **MRPL12**, impairing mitochondrial metabolic regulation. Collectively, these alterations drive cancer progression by enhancing **UBASH3B** tumor suppressive signaling. Figure created with BioRender.com

#### Tumor suppressor

UBASH3B plays an essential tumor-suppressive role through its regulation of tyrosine kinase signaling, immune signaling, and mitochondrial function [68–70]. UBASH3B functions as a negative regulator of receptor tyrosine kinase (RTK) signaling by inhibiting CBL, an E3 ubiquitin ligase responsible for the degradation of receptor-associated signaling proteins (Fig. 3) [48]. This inhibitory effect suppresses downstream oncogenic cascades and supports controlled cellular responses [48]. Further reinforcing its role in negative regulation of kinase signaling, UBASH3B inhibits BCR-ABL signaling in leukemic cells by binding to and dephosphorylating the BCR-ABL fusion protein, p210, its downstream signaling molecules, and its associated interactors, demonstrating a significant negative regulatory effect (Fig. 3) [68].

**Fig. 4:**
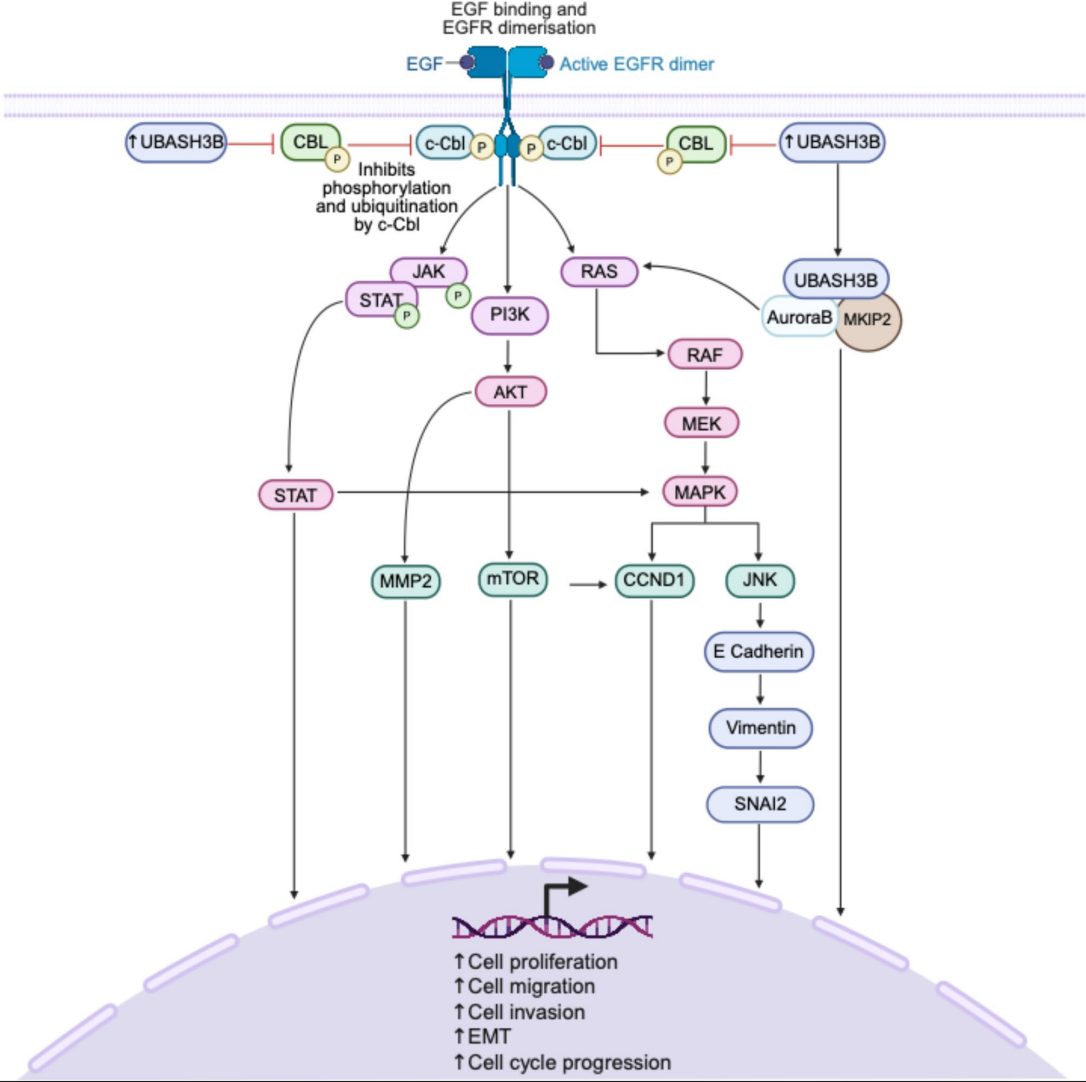
UBASH3B mediated oncogenic signaling and downstream effects. **UBASH3B** negatively regulates receptor tyrosine kinase (**RTK**) signaling pathways by inhibiting **CBL** activity, a key E3 ubiquitin ligase. This inhibition prevents the ubiquitination and degradation of receptor-associated signaling proteins, thereby modulating downstream signaling events. Activation of **RTKs** leads to the phosphorylation of **JAK3** and **STAT**, which triggers STAT-mediated transcriptional activation. **PI3K** activation results in the phosphorylation of **AKT**, promoting downstream targets such as **MMP2** and **mTOR**, which are involved in cell migration, invasion, and cell cycle progression. Activation of **RAS** promotes the **RAF-MEK-MAPK** cascade, leading to cyclin D1 (**CCND1**) activation and **JNK** signaling. **JNK** drives the epithelial-to-mesenchymal transition (**EMT**) through the downregulation of E-cadherin and the upregulation of mesenchymal markers, such as vimentin and **SNAI2**. **UBASH3B** forms a complex with Aurora B and **MKlP2**, promoting anaphase progression. Additionally, Aurora B can regulate the RAF-**MEK-MAPK** pathway by activating Ras. Together, these signaling pathways collectively contribute to cell migration, invasion, **EMT**, and cell **JAK3**cycle progression, processes central to cancer progression and metastasis. Figure created with BioRender.com

In lung adenocarcinoma (LUAD), UBASH3B is implicated as a tumor suppressor, supported by evidence of its role in mitochondrial regulation [69]. Additionally, low UBASH3B expression correlates with poor prognosis in LUAD [69]. Mechanistically, UBASH3B interacts with the mitochondrial ribosomal protein, MRPL12, dephosphorylating it at tyrosine 60, thus inhibiting LUAD development by driving mitochondrial metabolism reprogramming (Fig. 3) [69]. This post-translational modification maintains mitochondrial structure and supports the binding of MRPL12 to POLRMT, which is required for mitochondrial transcription and oxidative phosphorylation. Mutation of MRPL12 at Y60 (Y60A) mimicked this dephosphorylation, disrupting mitochondrial integrity and impairing oxidative phosphorylation, consequently promoting LUAD cell proliferation, migration, and invasion [69]. These findings highlight a novel tumor-suppressive axis mediated by UBASH3B through mitochondrial metabolic reprogramming [69].

Interestingly, a recent using multimodal sequencing technologies (spatial transcriptomics, scRNA-seq, bulk RNA-seq, and TCR sequencing) in hepatocellular carcinoma (HCC) patients receiving neoadjuvant nivolumab identified a signaling axis involving UBASH3B/NR1I2/CEACAM1/HAVCR2, which mediates immunosuppressive interactions among tumor-associated macrophages (TAMs), tumor cells, and T cells, fostering a pro-tumorigenic environment and resistance to immune checkpoint blockade [70]. This axis was found to be suppressed in immunotherapy responders and active in non-responders, implicating UBASH3B in immune evasion and suggesting its immunosuppressive regulatory role is context-dependent [70]. The presence of UBASH3B in this immune signaling loop supports its role in restraining immune activation under normal conditions, but also raises the possibility that its suppressive effects could be hijacked in tumors to evade immune surveillance [70].

Together, these findings demonstrate UBASH3B as a tumor suppressor through its role in immune regulation, inhibition of oncogenic kinase activity, mitochondrial homeostasis, and maintenance of proper cell division. However, these protective functions are highly dependent on the cancer cell type, and their loss or reversal can lead to oncogenic outcomes.

#### Oncogene

Although UBASH3B has clear tumor-suppressive functions, numerous studies have also identified it as an oncogenic driver, particularly by disrupting ubiquitin-mediated degradation of oncogenic proteins and enhancing pro-tumorigenic signaling pathways. UBASH3B is known to inhibit CBL, an E3 ubiquitin ligase that targets activated RTKs for degradation [48]. When this inhibitory role is disrupted, RTK activation triggers the phosphorylation of downstream components, initiating multiple signaling cascades including PI3K-Akt-mTOR and JAK-STAT pathways, which are frequently dysregulated in cancer (Fig. 4). By blocking CBL activity, UBASH3B allows persistent activation of RTKs, which leads to the phosphorylation of JAK3 and STAT, which triggers STAT-mediated transcriptional activation, which subsequently drives the transcription of genes involved in cell proliferation and survival (Fig. 4). Activation of RTKs. Simultaneously, RTK activation stimulates PI3K, resulting in the phosphorylation of AKT, promoting downstream targets such as MMP2 and mTOR, which are involved in cell migration, invasion, and cell cycle progression (Fig. 4). In parallel, RTK signaling also activates RAS, thus promoting the RAF-MEK-MAPK cascade, leading to cyclin D1 (CCND1) activation, which drives cell cycle progression (Fig. 4). Moreover, RAS activation induces JNK signaling, which facilitates epithelial-to-mesenchymal transition (EMT) by repressing epithelial markers like E-cadherin while upregulating mesenchymal markers such as vimentin and SNAI2 (Fig. 4). Collectively, these pathways converge to promote critical hallmarks of cancer, including enhanced cell migration, invasion, EMT, and uncontrolled cell cycle progression, highlighting the hypothetical interplay between UBASH3B regulation and downstream oncogenic processes (Fig. 4).

UBASH3B also plays a critical role in regulating mitosis. UBASH3B induces the spindle assembly checkpoint (SAC) progression by recruiting Aurora B to microtubules, promoting anaphase progression [71]. However, in the absence of UBASH3B, cancer cells expressing high levels of SAC proteins undergo mitotic arrest and cell death [51].

**Fig. 5:**
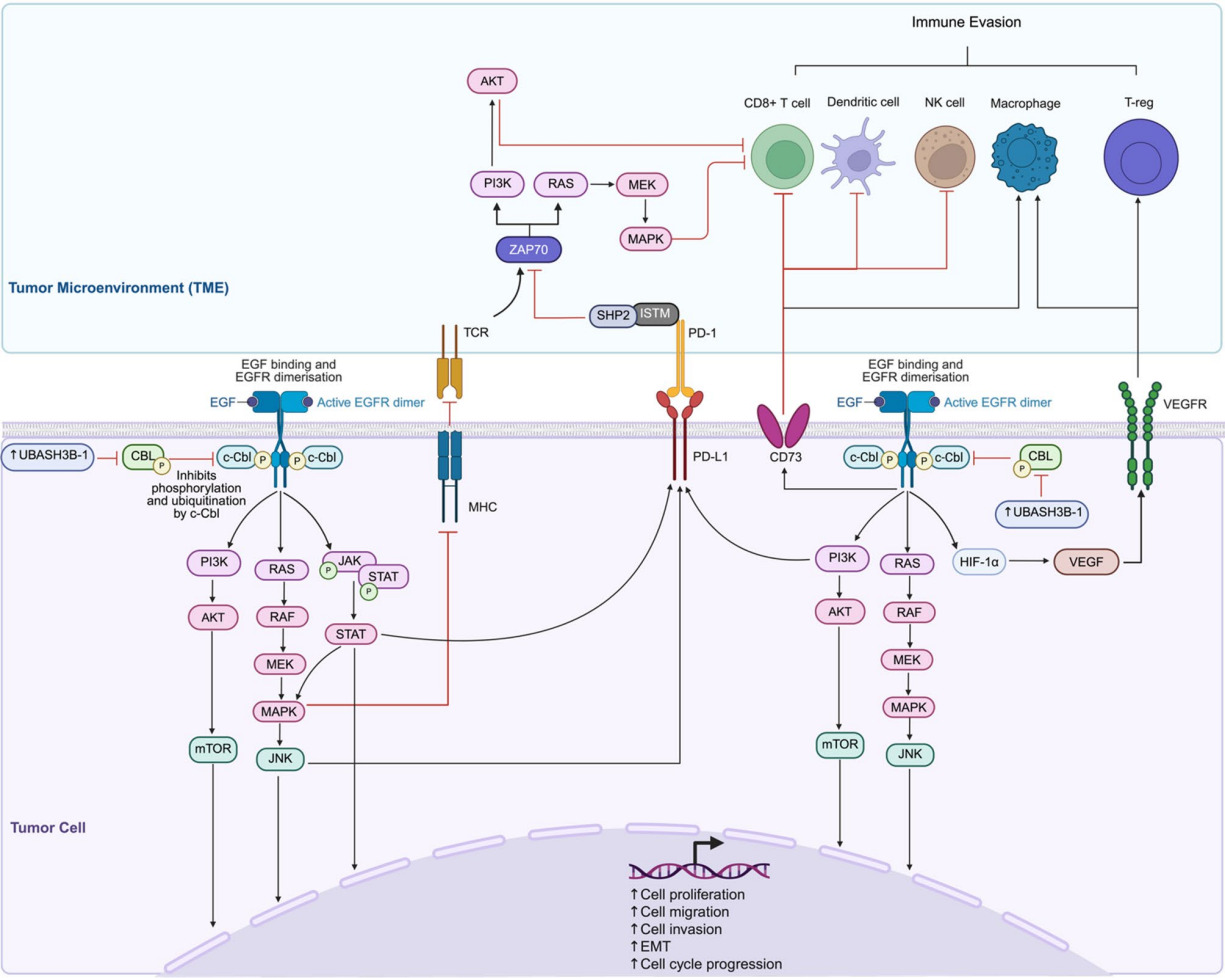
UBASH3B mediated immune suppression. **UBASH3B** inhibits the activity of the E3 ubiquitin ligase **CBL**, preventing degradation of receptor tyrosine kinase (**RTK**)-associated signaling proteins and promoting sustained activation of downstream effectors. **RTK** signaling leads to activation of **JAK3/STAT**, **PI3K/AKT/mTOR**, and **RAS/RAF/MEK/MAPK** pathways. The **MAPK** pathway suppresses immune recognition by inhibiting the expression of both major histocompatibility complex (**MHC**) molecules and T-cell receptor (**TCR**) signaling components. In tumor cells, **MAPK** hyperactivation downregulates **MHC** transcription, thereby reducing antigen presentation. In parallel, **EGFR** activation also upregulates **PD-L1** expression on tumor cells via downstream pathways such as **RAF-MEK-MAPK**, **PI3K/Akt**, and **JAK/STAT**, contributing to T-cell exhaustion and immune evasion. Upon binding of **PD-1** by its ligands, **PD-L1** expressed on tumor cells, the immunoreceptor tyrosine-based switch motif (**ITSM**) of **PD-1** is phosphorylated, leading to **SHP2** recruitment. **SHP2** inhibits **ZAP70**, thereby promoting downstream signaling cascades such as **PI3K/AKT/mTOR** and **RAS/MEK/ERK**. Activated **EGFR** signaling stabilizes the transcriptional activity of **HIF-a**, which then upregulates **VEGF**, which limits immune cell infiltration by fostering the accumulation of regulatory **T cells** (**Tregs**) and macrophages, all of which contribute to an immunosuppressive tumor microenvironment. Figure created with BioRender.com

Nick Carpino et al. (2004) identified UBASH3B as a negative regulator of TCR signaling [55]. Subsequent studies have reported UBASH3B to modulate T-cell receptor signaling by inhibiting T-cell activity and immune responses (Fig. 5) [72]. UBASH3B also exerts inhibitory effects in macrophages [59] and is expressed in dendritic cells and mast cells [73, 74], implicating the role of UBASH3B in a broader range of immune cell types. In prostate cancer, Wang et al. (2019) [75] demonstrated a strong correlation between UBASH3B expression and tumor-infiltrating immune cells, including B cells, CD4 + memory T-cells, regulatory T-cells, activated NK cells, M2 macrophages, dendritic cells, resting mast cells, and neutrophils, thus suggesting a role of UBASH3B in modulating the tumor immune microenvironment. A very recent study by Luo et al. (2025) [76] shed light on UBASH3B’s role in innate immunity. The study systematically analyzed innate immune cell barrier-related genes through differential expression profiling, pathway enrichment, and machine learning-based prognostic modeling in pancreatic cancer [76]. Notably, UBASH3B was identified as one of the top five core genes alongside ITGB6, COL17A1, MMP28, and DIAPH3 that significantly contributed to pancreatic cancer prognosis based on a random survival forest algorithm for patient outcome prediction [76]. The study proposed a dual role for UBASH3B in mediating both immunosuppression and drug resistance [76]. UBASH3B expression was significantly associated with immunosuppression in the TME, particularly through a negative correlation with NK-cell activation and positive correlation with other immune checkpoint molecules, including KL3C1, CBLB, KIR2DL1, and KIR2DL2 [76]. In drug sensitivity predictions, high-risk patients defined by UBASH3B-associated signatures exhibited resistance to erlotinib and oxaliplatin but retained sensitivity to 5-fluorouracil, thus proposing UBASH3B as a prognostic marker as well as a predictive biomarker for therapeutic stratification [76]. Similarly, another study in hepatocellular carcinoma identified UBASH3B as a central component of the immunosuppressive signaling axis involving NRI1I2, CEACAM1, and HAVCR2 [70]. Integrated spatial transcriptomics, single-cell RNA sequencing and TCR profiling revealed the UBASH3B-driven axis to be specially localized to tumor-associated macrophages (TAMs), tumor cells, and T-cells, within the TME, and contributed to the formation of a lipid-rich immunosuppressive niche that impaired T-cell cytotoxic reactivation and correlated with poor response to anti-PD1 therapy (neoadjuvant nivolumab therapy) [70]. Further, while non-responders exhibited higher UBASH3B expression and related immunosuppressive markers, responders demonstrated increased CD8 + T-cell infiltration [70], thus indicating UBASH3B as a mechanistic mediator of immune evasion and a potential predictive biomarker for nivolumab resistance.

In hematological malignancies and solid tumors as well, UBASH3B acts as an oncogene and is linked to poor prognosis. In acute myeloid leukemia (AML), UBASH3B contributes to myeloid proliferation and leukemogenesis through CBL inactivation and AML1-ETO-induced signaling [48]. In erythroleukemia, transcriptional activation of UBASH3B by FLI1 enhances ug resistance and leukemic progression while repressing anti-proliferative pathways [77]. Treatment with phorbol ester TPA was shown to induce UBASH3B expression, leading to degradation of PKCδ, a known suppressor of leukemogenesis [78]. Notably, RNAi-mediated depletion of UBASH3B reverses this effect, suppressing leukemic growth and overcoming TPA resistance [78].

Likewise, in prostate cancer, high UBASH3B expression is associated with poor clinical outcomes, including reduced overall survival [75]. UBASH3B has also emerged as a key oncogenic player in breast cancer progression, exhibiting distinct roles in both estrogen-receptor (ER)-positive breast cancer [79] and triple-negative breast cancer (TNBC) [47]. In both subtypes, high UBASH3B expression is associated with poor patient outcomes, including increased tumor aggressiveness, metastasis, and resistance to treatment [47, 79]. In ER-positive breast cancer, UBASH3B was associated with TP53 mutations and triggered resistance to tamoxifen, a commonly used endocrine therapy [79]. Mechanistically, UBASH3B plausibly interferes with ESR1 signaling, which encodes the ERa, and ESR1 itself can negatively regulate it [79]. The dysregulation of UBASH3B in ER-positive breast cancer thus compromises tamoxifen efficacy [79], leading to therapeutic resistance and disease progression. On the other hand, in TNBC, UBASH3B overexpression promoted cancer cell invasion and metastasis by downregulating the tumor-suppressive miR200a, a known suppressor of EMT [47]. Additionally, in HNSCC, LASSO-Cox regression analysis identified high UBASH3B expression as a marker of high-risk patients within a pyroptosis-based prognostic model, correlating with poorer overall survival [80]. These findings further support its oncogenic activity in epithelial malignancies.

## Potential therapeutic strategies

UBASH3B has emerged as a promising oncogenic target due to its involvement in a multitude of cancer-associated pathways, including PI3K/Akt/mTOR, MAPK, and JAK-STAT signaling. Additionally, its multidomain structure makes it an attractive yet complex target for therapeutic intervention. As mentioned above, the protein contains three key functional domains: the UBA domain facilitating ubiquitin-mediated interactions, the SH3 domain enabling binding to proline-rich motifs and adaptor proteins, and the histidine-based phosphatase domain (active site) responsible for its catalytic activity. Despite its potential, therapeutic strategies targeting UBASH3B are nascent, as it is a relatively novel protein and gene. However, recent studies have identified both small-molecule inhibitors and miRNAs as potential avenues for regulating UBASH3B activity (Table 1).

**Table 1:** Therapeutic strategies targeting UBASH3B and its domains. Small-molecule inhibitors target the protein, while MicroRNAs target the transcribed gene [47, 54, 63, 79, 81– 83]

Therapeutic	Name	Target
***Small Molecule Inhibitors***	PHPS1	Phosphatase domain (active site)
	Rebamipide	Histidine phosphatase domain
	Tetracycline derivatives	Periphery of the active site
	Sulfonated azo dyes	Active site
***microRNAs***	miR200a	3′ UTR of UBASH3B
	miR-148a-3p	3′ UTR of UBASH3B
***Chemotherapeutic Drug***	Cisplatin	UBASH3B

### Phosphatase domain inhibitors

UBASH3B is particularly a good target for small-molecule inhibitor development due to its distinct and targetable binding pocket within the active site in the histidine-based phosphatase domain of its structure [54]. Phenylhydrazono pyrazolone sulfonate (PHPS1), a Src homology-2-containing phosphatase (SHP2) inhibitor, has emerged as a potent small-molecule inhibitor for UBASH3B [54] and related phosphatases due to its ability to effectively target their active sites. PHPS1, a potent and selective inhibitor of SHP2 phosphatase activity, functions as a competitive inhibitor by binding to the phosphatase active site, thereby preventing substrate interaction [82]. Zhou et al. [54] revealed that PHPS1 also targets the histidine phosphatase domain of UBASH3B, thereby disrupting its regulatory role in TCR signaling. By competitively blocking UBASH3B’s phosphatase activity, PHPS1 could disrupt downstream oncogenic signaling pathways like Erk1/2 [82], offering a potential therapeutic approach for malignancies driven by UBASH3B overexpression. However, while these studies underscore the potential of PHPS1, further research is needed to optimize its specificity and evaluate its efficacy in preclinical models of UBASH3B-driven cancers.

Another potent inhibitor is Rebamipide, a drug traditionally used for treating stomach ulcers, which specifically binds to and targets the histidine phosphatase domain, effectively inhibiting UBASH3B enzymatic activity [81]. Additionally, structure-activity relationship studies revealed that Rebamipide derivatives also demonstrated strong inhibitory effects, suggesting potential for optimization [81]. Although Rebamipide exhibits low cell permeability, this limitation can potentially be addressed by employing liposomal packaging for efficient drug delivery [81].

High-throughput screening has further identified two additional classes of potential UBASH3B inhibitors: tetracycline derivatives and sulfonated azo dyes [83]. Tetracycline derivatives demonstrated weak inhibition, likely due to their peripheral binding to the active site of UBASH3B [83]. In contrast, sulfonated azo dyes, such as Congo red and Evans blue, exhibited stronger inhibitory effects by occupying the entire active site (phosphatase domain) of UBASH3B, making them more effective at blocking its activity [83]. Biochemical and functional assays demonstrated that these inhibitors could selectively inhibit UBASH3B proteins without affecting related phosphatases, disrupting their role in regulating TCR signaling [83]. This inhibition enhanced T-cell activation and immune responses, highlighting their therapeutic potential for immune modulation and cancer immunotherapy. While these small molecules represent promising leads for further targeting UBASH3B, in vitro and in vivo, studies are essential to validate their therapeutic potential and clinical relevance.

### miRNA-mediated regulation

In addition to small-molecule inhibitors, miRNAs represent another natural mechanism for regulating UBASH3B expression. miR-200a, a miRNA known to suppress invasion in various cancers, binds to the 3’ UTR of UBASH3B. miR-200a is often downregulated in TNBC, leading to increased UBASH3B expression and heightened oncogenic activity [47]. Likewise, UBASH3B is also targeted by miR-148a-3p in platelets, where increased miR-148a-3p expression decreases UBASH3B expression, thereby increasing platelet activation [63]. While these miRNAs offer significant potential for regulating UBASH3B, further investigation is warranted to evaluate their therapeutic efficacy in vivo.

### Drug repurposing

Interestingly, data mining from the Comparative Toxicogenomics Database (CTD) has also identified cisplatin as a potential therapeutic agent that could downregulate UBASH3B expression and enhance sensitivity to tamoxifen, presenting another potential treatment strategy [79]. Further studies are essential to validate the efficacy of cisplatin in inhibiting UBASH3B expression and to assess its potential as a combinatorial agent to restore chemotherapeutic drug sensitivity in cancer models.

Taken together, these findings highlight UBASH3B’s significance as a therapeutic target and emphasize the need for continued research to develop effective treatments targeting UBASH3B overactivity in cancer and other related conditions.

## Conclusion

In this review paper, we have summarized current knowledge on UBASH3B, a multifunctional protein tyrosine phosphatase with a pivotal role in mammalian development and cellular signaling, with emerging significance in cancer and other diseases. UBASH3B modulates several pathways by dephosphorylating key kinases such as EGFR, ZAP70, Syk, and MRPL12, therby modulating key oncogenic and immune-related pathways. Its ubiquitous expression across all cell types and functional involvement in both immune regulation and tumor progression underscore its significance as a central node in intracellular signaling. Importantly, UBASH3B acts as a dual regulator of cancer progression and immune modulations, with its role varying depending on the cellular context. UBASH3B functions either as a tumor suppressor in some cancers, such as TCR and LUAD, by inhibiting oncogenic kinase pathways, and enhancing mitochondrial metabolism, while paradoxically functioning as an oncogene in other cancers such as leukemia, prostate, breast, and head and neck cancers. These dichotomous functions reflect the complexity of the biology of UBASH3B and highlight its translation potential. Altogether, UBASH3B presents as a promising biomarker and therapeutic target. Understanding its dual functionality will be key to leveraging its role in precision oncology and in designing combination strategies that target both tumor-intrinsic and immune-mediated mechanisms of resistance.

## Data Availability

No datasets were generated or analysed during the current study.
